# Gallstone Ileus–Clinical and therapeutic aspects

**Published:** 2010-11-25

**Authors:** M Beuran, I Ivanov, MD Venter

**Affiliations:** * ‘Carol Davila’ University of Medicine and Pharmacy,Bucharest Romania; ** Clinical Emergency Hospital, Bucharest Romania

**Keywords:** gallstone, ileus, pneumobilia, surgery

## Abstract

Gallstone ileus represents a rare (0.3–0.5%)[15], but serious complication of a common illness–the gallbladder lithiasis and the incidence of this fascinating disease has remained the same over the years. The main actual characteristics of this pathology are the age over 65, the female gender (men/women ratio 1/5:1:10–due to the high rate of vesicular lithiasis) and the under 50% diagnostic established preoperatively. The frequency of gallstone ileus recurrence is of 4,7–5%.

In this article, we discuss the pathogenesis of this illness presenting all the mechanisms described in the medical literature. The Rigler triad found at the abdominal CT–scan generally established the diagnosis. Still, in 25% of the cases we have a misdiagnosis because of the underestimation of the size of the gallstone.

Finally, the treatment of gallstone ileus has had major changes from the past. We described the endoscopic and laparoscopic approach, which represents the modern treatment of this disease. Despite these diagnostic and therapeutic possibilities, the mortality remains high and the common causes are associated comorbidities and late presentation to the physician.

Gallstone ileus represents a rare (0.3–0.5%)[1], but serious complication of a common illness–the vesicular lithiasis, which has a prevalence of 10% in the USA and Western Europe [2]. In these days, when the laparoscopic cholecystectomy represents the ‘Golden Standard’, the incidence of this fascinating disease has remained the same. The morbidity and mortality rate still have a high percentage. These are due to the old age of the patients, the associated pathology (usually severe), late admittance in the hospital and the delayed therapeutic treatment.

The incidence of the gallstone ileus has been maintained constant over the years: 30–35 cases/1.000.000 admissions in the hospital over a period of 45 years (Kurtz cit.Moberg–3), 0,9/100.000/year [3]. In the cases of laparotomies for small bowel obstruction, the incidence is about 2–3% (4, Kasahara cit.^5^). This disease represents almost 25% of the unstrangulated small bowel obstructions in the patients over 65 years old.

The youngest patient reported, was a case of a 13 year old [7], and the oldest (Japanese articles) of 91 years old

Thomas Bartholin described the first case of gallstone ileus in 1654, in a necropsy study.

- 1841–Bonnet presented the first case of duodenal obstruction [1] (gallstone impaction in the duodenal bulb–later known as Bouveret syndrome).
- In 1890, Courvoisier published the first 131 cases of gallstone ileus, secondary to gallbladder–duodenal fistula [9,10,11,12]; 125 cases were operated on with a mortality rate of 44%.
- 1896–Leon August Hoffmann Bouveret established the first preoperatory diagnostic.[1,11].
- 1914–von Wagner published 334 cases of gallstone ileus (cit.^6^,^12^)
- 1925–British Medical Association presented 3066 cases of intestinal obstructions, 28 being caused by gallstones [12].
- 1932–Henry Huberrt Turner describes colonic obstruction by a gallstone [1]: a gallstone of 7,5 x 5,7 cm, having a circumference of 17,8 cm and the impaction at the transverse colon

The main actual characteristics of this pathology are: the age over 65, the feminine gender (men/women ratio 1/5:1:10–due to the high rate of vesicular lithiasis) and the under 50% diagnostic established preoperatively [2,7,8,14,15].

The frequency of gallstone ileus recurrence is of 4,7–5%[16,17]. Characteristic is an anterior episode of gallstone bowel obstruction. Just 10% of the patients need surgical reintervention due to recurrent billiary symptoms [14]. It is associated with a high rate of mortality 20% [17], being defined as an enteral mechanical obstruction due to an endoluminal gallstone present at the time of the first operation or due to a new gallstone present in the bowel through an untreated bilio–enteral fistula (Ulreich, Levin in [18]). Faceted or cylindrical gallstones can be predictive for recurrent ileus. Buetow thinks that the majority of recurrent gallstone ileus appear in the first 30 days from the initial operation and the site of the obstruction is the ileum in most cases. Our cases confirm what Buetow found.

## Mechanism

The most frequent mechanism of appearance is represented by the migration of a gallstone through a gallbladder–duodenal fistula (68%, 96,5% Japanese patients–8), but gallbladder–jejunal fistula, gallbladder–colonic fistula (5–25%–9,20), gallbladder–duodenal–colonic(2,5%), gallbladder–gastric (Clavien, Rodriguez Sanjuan, Glenn in [21]), common billiary duct–duodenal, duodeno–left hepatic billiary duct [1,22], have also been reported.  The classic gallstone ileus is preceded by an acute cholecystitis episode, inflammation and gallbladder–bowel adhesions (usually at the level of the fundus of the gallbladder), which, associated with the ischemic effect and local pressure exercised by the gallstone, facilitates the creation of a bilio–enteral fistula (the decrease in the arterial, venous and lymphatic flow associated with the increase in the intravesicular pressure). Sometimes the cystic obstruction can be realized as well [16]. The gallbladder becomes scleroatrophic, not functional in most cases.

The size of the gallstone plays an important part in the appearance of the bowel obstruction, the majority of the authors saying that gallstones of over 2,5 cm realize the bowel impaction (in the absence of a digestive pathology which can determine stenosis–spasm, adhesions, Crohn disease).

Usually, the gallstone impaction is realized in the terminal ileum (65%) and the ileocecal valve (the most narrow part of the small bowel, with lower peristaltic movement–[9,14,23]). The potential reactive substances in the bile can interact with the epithelium cells of the bowel and can induce the impaction of the gallstone associated with lesions of the mucosa (Chipman in [8]).

The duodenal obstruction appears in rare cases (3–10%), being described by Bouveret in 1896; seldom, the gallstone can block the entrance of a Meckel diverticulum (Nakamoto–24. Emparan), the sigmoid colon (Mastin–[6]) or the appendix (Mehrotra in [11]). The sigmoid colon is the most frequent location of the colon obstruction (due to the stenosis produced by diverticulitis), being described by Reisner [14] in only 4% of the cases.

In rare occasions, the gallstone can create a fistula between the common billiary duct and the gastro–intestinal tract.

There have been cases of bowel obstruction caused by gallstones, without the intraoperatory finding of a bilio–enteral fistula [26]. The explanation resides in the migration of a gallstone through the Vater papilla followed by the ‘in situ’ growth. This may happen in the event of a stenosis of the small bowel. Yoshida and co. published a case of gallstone ileus determined by the migration through the Vater papilla.

Lasadro [18] Lindsey and Warner [28] presented the gallstone ileus postcholecystectomy, the gallstone passing through the CBD (after sphyncterotomy) or from a duodenal diverticulum. Saedon [29] presents a case that appeared after 24 years of postcholecystectomy, in a patient with jejuno–ileal diverticulosis.

Draganic [28] and Dittrich [30] published two cases of bowel obstruction, which describe the migration of a gallstone through the jejunal wall after being lost intraoperatorily, due to a difficult laparoscopic cholecystectomy. These are the first cases produced by this mechanism. There is just one more case published, of a subocclusion resolved in a conservative manner (Wills –[31]). Later, other authors presented cases produced by the same mechanism [32]. Habib [33] published a case of a gallstone ileus after 8 years of postcholecystectomy, as a result of a gallstone lost intraoperatorily, that migrated transepiplooicly, and eroded the apex of a Meckel diverticulum. Afterwards, the gallstone moved in the bowel determining obstruction. The gallstone was mobilized cranially and removed through the large base of the Meckel diverticulum.

The association of the Mirizzi syndrome with the cholecysto–enteric fistula was first suggested by Beltran [34]. Subsequently, cases of gallstone ileus of type Ⅳ Mirizzi syndrome, associated with a cholecysto–colic fistula were cited in the medical literature.

Iatrogenous gallstone ileus can appear after ERCP with sphyncterotomy (Allen [16]) and in the absence of the gallbladder.

Patients with a prolonged evolution of the Crohn disease present a particular risk of developing gallstones, because of the overall modification in the entero–hepatic circuit of the bile salts (secondary of terminal ileitis) and the solubility of the cholesterol [35] especially after an ileum resection. The modified peristalsis and the high content of the bile in the bilirubine levels, also contribute to this.

The average size of an obstructive gallstone in patients with Crohn disease is lower than the ones in ‘classic’ described cases: 2.5 cm (1,5–3cm) opposed to 4,5 cm(2–9 cm). This can be explained by the enteral fibrous stenosis [5].

Colonic gallstone ileus is the most frequent in the case of a stone passed through a gallbladder–colon fistula. The colonic gallstone ileus has also been described in a gallbladder–duodenal fistula (Moller 1913, Harris, McNamara and Dardinsky 1947, Buetow, Glaubitz and Crampton 1963 [13]) or a CBD–duodenal fistula (Shore, Jacob and Cannon 1953). Holm-Nielsen and Linnet–Jepson think that it occurs when many small gallstones come together to form a bigger one, and Haffner, Semb and Aakhus describe the growth of the gallstone due to the accumulation of faeces around it, as a possible cause [13].

## The diagnostic

It is difficult, due to all the non–specific symptoms. An early diagnostic can reduce the mortality rate. Cooperman’s studies found that there is an average period of 7 days from the start of the symptoms until the admittance in the hospital (independent prognostic factor) and 3.7 days until the surgical intervention [7]. A characteristic of the gallstone ileus is a delayed period of 7 to 10 days from the symptoms debut until the surgery.

In 50% of the cases, the history of the patients reveals an old suffering due to gallstones. The symptoms may suggest a small bowel obstruction, and besides pain, we can find nausea, vomiting and sometimes haemetemesis (because of duodenal erosions caused by the migration of the gallstone).

There are three clinical types of this disease:

- Acute–it corresponds to the ‘classic’ gallstone ileus;
- Subacute–presenting as a bowel subocclusion;
- Chronic (Karewsky syndrome)–repeated episodes of pain due to the passage of the gallstone into the bowel (Rodriguez Hermosa 38).

Hildebrandt [39] described three clinical types: blocked (50%), remittent (30%) and peritonitis (20%).

The clinical preoperative diagnostic can be established on the Mordor Triad: history of gallstones, clinical signs of cholecystitis and bowel obstruction suddenly installed, with an interrupted progress.

A positive diagnostic is usually based on clinical examination (small bowel obstruction), plain abdominal radiography and abdominal CT scan.

**The plain abdominal radiography** highlights:The Rigler triad: pneumobilia, ectopic radio opaque gallstone, intestinal distension (Rigler, Borman, Noble–1941 [40]);

The presence of the Rigler triad in the plain abdominal radiography varies between 17–87% [16,41], if present, 2 out of 3 signs, are considered sufficient to establish a diagnostic. Pneumobilia may also be present in the case of a bilio–digestive anastomosis (CBD–duodenal anastomosis is the most frequent), endoscopic sphyncterotomy and a wide Oddi sphincter.

- The Rigler tetrad: the Rigler triad +/– a shift in the position of the gallstone observed in a previous examination (Rigler in [2]).
- The presence of two fluid–filled loops in the right upper quadrant, the medial one corresponding to the duodenal bulb and the lateral one to the gallbladder (due to the presence of air). This represents the fifth radiological sign described by Balthazar and Schechter [42].

**The abdominal CT scan** can highlight a:

- Pneumobilia
- Occlusive bowel
- Ectopic gallstone/gallstones in the bowel lumen
- Chronic colecystitis–changes in the gallbladder wall
- Cholecysto–enteral fistula+/–local inflammation
- The size of the gallstone–especially in the transition zone between the distended intestine and the stenotic bowel

Yu and co's diagnostic criteria for gallstone ileus at the CT scan are small bowel obstruction, ectopic gallstone, gallbladder modifications (pneumobilia, inflammation of the gallbladder wall, edema of the gallbladder wall). In this prospective study, the authors concluded that the contrast CT examination has 93% sensitivity, 100% specificity and 99% accuracy for the diagnosis of gallstone ileus.

Today, the CT scan represents the optimal way to diagnose the gallstone ileus. It can establish the site and nature of the obstruction [60]. The Rigler triad found at the CT scan represents a special diagnosis value [52]. Still, in 25% of the cases we have a misdiagnosis because of the underestimation of the size of the gallstone [60].

By using MDCT, Lassandro and Pickhardt [18,44] were able to diagnose the presence of a gallstone inside the bowel lumen, the number of gallstones and their dimension. They were able to put an early diagnosis of gallstone ileus preventing the changes made by the gallstone impaction. Furthermore, once the bilio–enteral fistula is seen, the surgeon will be able to decide on the opportunity of performing the cholecystectomy (in wide fistula that associates the presence of residual gallstones). The MDCT can offer crucial information in choosing the appropriate treatment.

### The abdominal ultrasound

It can diagnose residual gallstones, the presence of bilio–digestive fistula, gastric distension in the Bouveret syndrome, even the gallstone impacted in the bowel.

The association of the abdominal ultrasound with the plain abdominal radiography increases the diagnosis sensitivity up to 74% (Ripolle in [15,41] Oikarinen, Lassandro in [11]). The ultrasound can diagnose pneumobilia and ectopic gallstone.

The MRCP proves the pneumobilia, but it has a decreased value in the diagnosis of gallstone ileus. Pickhardt [47] thinks that the MRCP can appreciate the correct size of the gallstone.

Rarely, the exploratory laparoscopy is used for the diagnosis.

### The Treatment

The purpose of the treatment of the gallstone ileus represents the extraction of the gallstone, this way solving the bowel obstruction.

The surgical intervention has to be preceded by a volemic and hydro–electrolytic rebalance and the correction of the preexisting pathology.

Clavel stressed that the gallstone ileus is an umoral disease before being a surgical one. It is one of the most serious umoral imbalances. He states that the treatment of this acute bowel obstruction is not based on the surgical technique, but on a surgical strategy issue. The surgical timing must be chosen properly.

**The surgical treatment** varies between the classic enterolythotomy and the ‘one stage’ method. Each technique has its supporters, indications and contraindications depending on the general status of the patient. The risks of the classic surgical intervention required new therapeutic treatments: interventional endoscopy, ESWL procedures (extracorporeal shock wave lithotripsy) ultrasound directed (Clavien in [1,18]; Meyenberger in [15,16,17]) and laparoscopic surgery. These procedures are still waiting for the test of ‘time’ in order to be correctly used and approved.

Sometimes, ‘through gastric washout followed by intestinal continuous drainage’, the gallstone ‘may be eliminated via natural ways’ [48].

The classical surgical procedures are represented by:

1. Enterolithotomy: It requires an enterothomy (most frequently of 15–20 cm proximally to the obstruction site, after the gallstone mobilization upstream), the gallstone extraction is followed by an enteroraphy in one plane or two. One can notice the immobility of the gallstone. Above it, the bowel is distended. In the case of a gallstone mobilization, a litothripsy with a clamp may be applied through a little opening near it [48]. The distended bowel, leads the surgeon to the site of the obstruction. It is indicated to mobilize the gallstone downward (very efficient, but rarely possible) or, in the most frequent cases, upward, so that the enterothomy may be performed in a safe zone, where the lesions of the mucosa and/or the disorders in the local microcirculation are absent or as few as possible. The enterothomy will be done longitudinally, on the antimesenteric margin and it is followed by the extraction of the gallstone and the manual exploration of the entire small bowel (to find a possible remnant gallstone) and closed by a longitudinal or transverse enteroraphy. The exploration of the cholecysto–duodenal zone is not indicated in the elderly patient with a severe general status [4]. This also represents the conduct that is accepted and performed in the General Surgery Clinic of the Clinical Emergency Hospital, Bucharest.
  The Kopel maneuver (pushing the gallstone through the Bauhin valve with the help of a clamp) is forbidden, due to the possibility of causing damage to the bowel wall [1].
  This method presents the following advantages (defined for the emergency surgery): quick fix in the severe cases; simple as technique; fast. The disadvantages are the risk of recurrent gallstone ileus due to the ‘silent second stone’; the persistence of biliar symptoms or due to the bilio–digestive fistula (acute cholecystitis, recurrent colangitis); the high risk of gallstone carcinoma in the presence of a bilio–intestinal fistula (Berliner and Burson in [1,7,14]); malabsorbtion (cholecysto–colonic fistula).
2. Segmental enterectomy–in case of perforation or severe ischemic lesions
3. ‘One stage procedure’ –enterolithotomy (proximally to the obstruction site), colecystectomy and repair of the bilio–digestive fistula. It ensures a definitive treatment, avoiding a new surgical intervention. A great disadvantage–high morbidity and mortality rate [14,49], due to the complexity of the intervention and the increased time of it. Indications: acute cholecystectomy, gangrenous gallbladder, residual gallstones when the laparotomy is performed [50], patients with low risk rate [16].
4. Two stage procedure–enterolithotomy initially followed at 4–6 weeks by cholecystectomy and repair of the bilio–digestive fistula. Criteria are: age, hydro–electrolytic imbalance, associated chronic pathology, residual gallstones and an acute inflammatory process at the fistula level (Adorni, Raf, Warshaw in [9,51]). According to Zaliekas [41], this method is suitable for young patients, who have a higher risk of subsequent billiary complications and in the case of recurrent biliary symptoms.

### Laparoscopic procedures

They do not represent the therapeutic ‘golden standard’ [41], because of the difficult examination of the distended bowel, finding the gallstone, increased time of the surgery and the need for specialized trained surgeons in advanced and emergency laparoscopy [15,52].

Until 2001, there were three studies regarding the use of laparoscopy in the treatment of gallstone ileus: Montgomery [53], Franklin [54], Sarli [55]. The development of the laparoscopic surgery imposed the need to use this method more frequently.

The enterolithotomy may be performed laparoscopically [21] or laparoscopically assisted [22, 60], when, after finding the gallstone, the bowel segment is pulled out through a small laparotomy, the gallstone is mobilized backward and the enterolithotomy is performed.

In a retrospective study (32 patients with gallstone ileus with laparoscopic enterolithotomy and classic surgery, between 1992 and 2004; 19 laparoscopic; 2 conversions), Moberg and Montgomery [3] conclude that laparoscopically assisted enterolithotomy may be recommended for the diagnostic or therapeutic purposes.

The majority of authors agree that the optimal solution is represented by enterolithotomy [3,4,8,15,56,57]. The presence of a big gallstone in the gallbladder (ultrasound finding) would call for the one stage procedure to avoid the recurrent gallstone ileus.

Tan and co. [57] decided that both methods are safe, with zero mortality rates, but considers that the enterolithotomy is the best choice.

### Interventional endoscopy

Represents the best treatment for the patients with increased risk rate. 

The endoscopic extraction of the gallstone (impacted in the duodenum or in the colon) is a possible modern procedure. It can be associated with the endoscopic lithotripsy [58].

Other methods are:

- Laser colonic lithotripsy with a tissue recognizing system [20];
- ESWL (Clavien in [1,51]);
- ESWL plus Argon plasma coagulation [59];
- Nd YAG laser lithotripsy/Holmium YAG laser [60];
- Endoscopic electro–hydraulic lithotripsy (Bourke in [15,20]).

We have to mention that the lithotripsy may produce distal gallstone ileus [60].

### The medical treatment

May be applied when the gallstone is under 2cm, radiologically measured; 14.2% of the Japanese patients have been treated successfully this way [8]. Sometimes the passing of the stone may occur after the endoscopic attempt of extracting it.

Patients who have had an enterolithotomy must be controlled with the help of the ultrasound. We have to take into account the need to perform ESWL/chemical  dissolution of the gallstones in patients who have an increased risk and gallstones are present after the enterolithotomy [51].

The mortality rate varies between 0–25% [1,8,11,14,15,16,22]. The principal causes are associated with comorbidities and late presentation to the physician.
